# Asparagine Synthesis during Tobacco Leaf Curing

**DOI:** 10.3390/plants8110492

**Published:** 2019-11-11

**Authors:** Lucien Bovet, Cecilia Cheval, Aurore Hilfiker, James Battey, Delphine Langlet, Herve Broye, Joanne Schwaar, Pierrick Ozelley, Gerhard Lang, Nicolas Bakaher, Helene Laparra, Simon Goepfert

**Affiliations:** Philip Morris International R&D, Philip Morris Products SA, CH-2000 Neuchâtel, Switzerland; cecilia.cheval@pmi.com (C.C.); aurore.hilfiker@pmi.com (A.H.); James.Battey@pmi.com (J.B.); delphine.langlet@pmi.com (D.L.); herve.broye@pmi.com (H.B.); joanne.schwaar@pmi.com (J.S.); pierrick.ozelley@pmi.com (P.O.); gerhard.lang@pmi.com (G.L.); Nicolas.Bakaher@pmi.com (N.B.); helene.laparra@pmi.com (H.L.); simon.goepfert@pmi.com (S.G.)

**Keywords:** asparagine, tobacco, curing, senescence, asparagine synthetases, nitrogen assimilation

## Abstract

Senescence is a genetically controlled mechanism that modifies leaf chemistry. This involves significant changes in the accumulation of carbon- and nitrogen-containing compounds, including asparagine through the activity of asparagine synthetases. These enzymes are required for nitrogen re-assimilation and remobilization in plants; however, their mechanisms are not fully understood. Here, we report how leaf curing—a senescence-induced process that allows tobacco leaves to dry out—modifies the asparagine metabolism. We show that leaf curing strongly alters the concentration of the four main amino acids, asparagine, glutamine, aspartate, and glutamate. We demonstrate that detached tobacco leaf or stalk curing has a different impact on the expression of asparagine synthetase genes and accumulation of asparagine. Additionally, we characterize the main asparagine synthetases involved in the production of asparagine during curing. The expression of *ASN1* and *ASN5* genes is upregulated during curing. The *ASN1-RNAi* and *ASN5-RNAi* tobacco plant lines display significant alterations in the accumulation of asparagine, glutamine, and aspartate relative to wild-type plants. These results support the idea that ASN1 and ASN5 are key regulators of asparagine metabolism during leaf curing.

## 1. Introduction

Senescence is the terminal phase of the leaf development process. During senescence, plant cells initiate a range of responses that include a decline in photosynthetic capacity, reduction in chlorophyll and water content, transcriptional activation of the senescence-associated genes (*SAGs*), degradation of cellular structures, and, eventually, re-assimilation and remobilization of mineral nutrients and nitrogen-containing molecules from the leaves. In plants, nitrogen is essential for growth and development. Nitrogen re-assimilation and remobilization from senescing leaves to reproductive organs and growing upper leaves is an adaptive mechanism critical for their survival.

A major aspect of leaf senescence is the metabolic transition from anabolism to catabolism. During their growth and development, plants can primarily assimilate inorganic nitrogen present in soil nitrate, which is provided by fertilization or bacterial nitrification, for example [1]. Inorganic nitrogen is assimilated in the form of nitrate (NO_3_^−^) and ammonium (NH_4_^+^). The pools of NO_3_^−^ and NH_4_^+^ are usually stored in the vacuoles of leaf cells [2] and in photosynthetically active tissues. The nitrogen from NO_3_^−^ and NH_4_^+^ can be incorporated into amino acids and stored in a range of proteins, including the Rubisco, chloroplastic glutamine synthetase (GS) GS2, and vegetative storage proteins (VSP) [3,4]. During senescence, free amino acids and nitrogen are released from the breakdown of proteins by proteases. Nitrogen is re-assimilated preferentially into specific amino acids, which are loaded into the phloem for long-distance transportation to reproductive organs and younger tissues, namely sink tissues [5]. Over the last decades, large-scale metabolomic analyses have been conducted to determine how nitrogen is re-assimilated and then remobilized into sink tissues during leaf senescence [6,7,8,9,10,11]. These analyses have demonstrated that the total amino acid concentration decreases in leaves and increases in the phloem sap [6]. In addition, the amino acid spectrum changes significantly and progressively during senescence [12]. The most abundant forms of amino acids transported are asparagine (Asn), aspartate (Asp), glutamic acid (Glu), and glutamine (Gln) [13]. The timing of production, nature, and concentration of these amino acids in senescing leaves vary among plant species. In *Arabidopsis* leaves, for example, the Glu and Asp content rises rapidly during senescence and then decreases, whereas the Gln and Asn content increases progressively with age [10,14]. This suggests an active interconversion of Asp to Asn and Glu to Gln during leaf senescence. Other studies on tobacco have reported a decrease in Gln and Asn content during senescence, which suggests that nitrogen is re-assimilated and then remobilized rapidly to sink tissues [7]. Overall, most of these metabolomic analyses strengthen prior evidence showing that nitrogen is re-assimilated into Gln and Asn, which are currently described as the main mobile nitrogen-rich amino acids to be transported to other organs. Only few senescence-related enzymes that allow nitrogen re-assimilation into amino acids have been reported so far; however, their mechanism is still poorly understood. These include glutamine synthetase, glutamine oxoglutarate aminotransferase (GOGAT), asparagine synthetase, glutamate dehydrogenase, and threonine deaminase [15,16]. With the discovery that acrylamide—a human carcinogen—can be formed in carbohydrate-rich food (e.g., potato) as a result of thermal degradation of Asn during the Maillard reaction [17], many research studies have been conducted to understand the role of asparagine synthetases and other regulatory components in the biosynthesis and degradation of Asn.

Leaf senescence can be influenced by intrinsic and extrinsic factors, including age, reproductive growth, endogenous hormones, light, temperature, salinity, and pathogens [18]. In addition, detachment of plant leaves from the stem can artificially and rapidly trigger leaf senescence and is, hence, the method of choice for studying senescence in laboratory conditions [19]. Many research studies reported that senescence mechanisms in naturally senescing leaves and detached leaves are at least, partially similar [20,21,22,23]. Execution of senescence in detached tobacco leaves is observed during curing—a process that allows tobacco leaves to dry out in ventilated or heated barns or under the sun, for a period of one to nine weeks. Besides being a model plant in biological research, tobacco is an important economic crop, and curing of detached tobacco leaves has a strong impact on the quality of the final tobacco product [24,25].

The mechanism of regulation of nitrogen assimilation during tobacco air curing remains elusive. Here, we examine our recent discoveries that demonstrate how the air curing process modifies asparagine metabolism and, hence, nitrogen re-assimilation in burley tobacco leaf tissue and discuss hypotheses on the mechanisms behind the observed modifications. Importantly, we found that the production of Asn dramatically increases during curing. Hence, regulation of the production of Asp, Glu, and Gln, which are part of the asparagine synthesis pathway, is also altered in air cured leaf tissues. By resolving the expression profile of asparagine synthetase genes (*ASN*) in tobacco during curing, we identified four genes *ASN1-S*, *ASN1-T*, *ASN5-S*, and *ASN5-T* that are upregulated during leaf curing. Furthermore, we compared two methods of curing—detached leaf versus stalk curing—and observed robust differences in the assimilation of Asn between the two processes. Using a genetic approach, we eventually showed that Asn production during air curing results from the specific activity of asparagine synthetases encoded by *ASN1* and *ASN5*. During smoking, acrylamide is produced as a result of the Maillard reaction that occurs between asparagine and reducing sugars. A better understanding of the asparagine synthesis pathway in plants during curing will allow better control of the acrylamide accumulation in smoking or heated tobacco products. Consequently, the fundamental knowledge gained in this work creates a framework from which new tools can be developed for managing the reduction of acrylamide in tobacco products.

## 2. Results

### 2.1. Air Curing Modifies the Asparagine, Aspartate, Glutamine, and Glutamic Acid Content in Burley Tobacco Leaves

**Figure 1:**
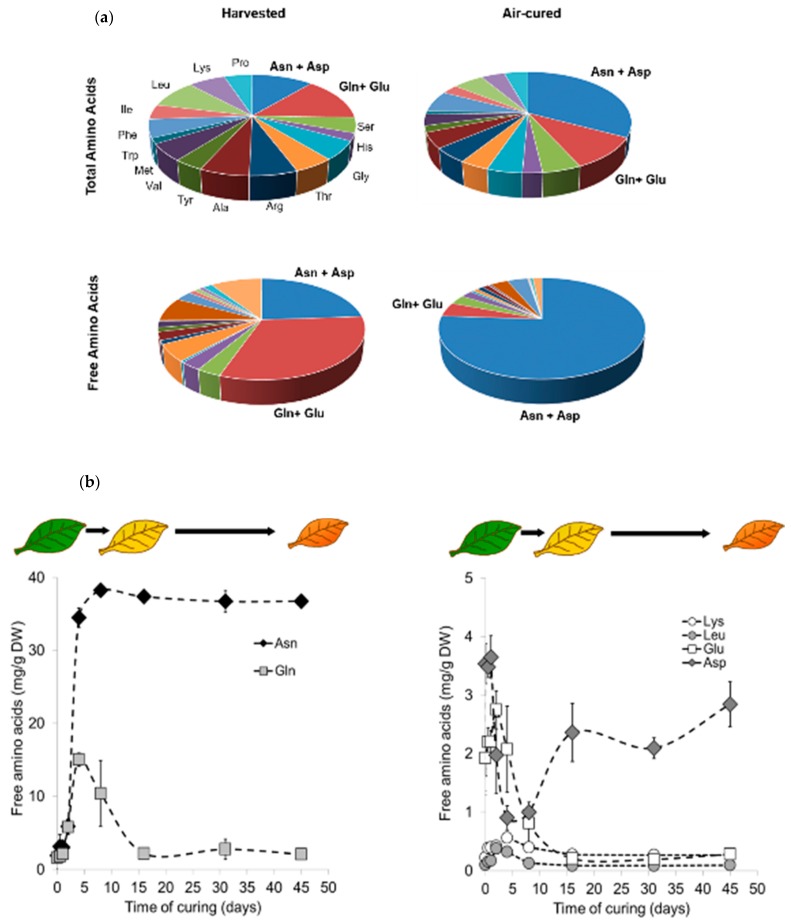
(**a**) Chart of total and free amino acid distribution in mature leaves of burley tobacco at harvest and after air curing. Each sector represents the frequency distribution (mg/g dry weight [DW]) of amino acids in the total amino acid fraction, and after hydrolysis (free amino acids) when the leaf tissue is harvested and air cured (45 days post-harvest). Data were collected from three biological replicates (n = 3). (**b**) Time course of the air curing effect on the accumulation of asparagine and glutamine (left panel) and aspartate and glutamic acid (right panel) in tobacco burley leaves. The free amino acids were quantified at different time points during air curing. Data were collected from three biological replicates (n = 3). Each data point represents the mean free amino acid content (in mg/g of dry weight [DW]). Vertical bars are standard deviation (SD).

We evaluated the importance of air curing on the amino acid content in burley tobacco leaf tissues. Burley tobacco plants were grown in the field for about 12 weeks, after which the leaves were detached from their stalk and cured on a string under dim light in ventilated barns for 45 days to dry out. We compared the composition of total (amino acids bound in protein units) and free amino acids at harvest and end of curing (Figure 1a and Table S1). We quantified nitrate and alkaloid levels as a control in this experiment (Figure S1a). Relative to the accumulation at harvest, the nitrate levels increase after air curing, whereas the total alkaloid content decreases slightly, which is consistent with previous observations [26,27]. The total amino acid content decreases significantly in air cured tobacco leaves compared with harvested leaves (Figure S1b). In contrast, we observed a 5.2-fold significant enrichment of free amino acids at the end of air curing, although the free amino acid content remains very low at harvest and at the end of the curing process (<50 mg/g dry weight; Figure S1b).

Senescence-associated proteolysis is tightly controlled through transcriptional regulation. Transcription of genes encoding proteases is strongly upregulated during leaf senescence and correlates with proteolytic alterations [28,29]. In this study, we identified 50 significantly upregulated genes coding for proteases (reported in Table S2 on the basis of their putative function) after 48 h of curing. This observation strongly suggests that proteases are effectively active during curing and may contribute to the accumulation of free amino acids or peptides. Indeed, quantification of accumulated peptides revealed a global increase in dipeptide levels during curing, which likely results from proteolytic activities (Figure S2). Similarly, we expected an increase in the global free amino acid pool. Although the concentration of some amino acids increases significantly, we mostly observed a strong increase in Asn and Asp levels, as shown in Figure 1a. Therefore, the main senescence-activated proteases have probably only a minor or indirect implication in Asn accumulation during curing.

When we analyzed the amino acid composition of harvested and air cured tobacco leaves, we noticed that the major differences lay in the concentrations of a few amino acids—Asn, Asp, Glu, and Gln (Figure 1a). It should be noted that, for total amino acid analysis, the method of peptide hydrolysis used in this study did not discriminate between Asn and Asp or Gln and Glu (see Material and Methods). At harvest, Asn and Asp represents 11% and 24% of the total and free amino acid content, respectively, while Gln and Glu represents 14% and 32% of the total and free amino acid content, respectively. Although the proportion of Gln and Glu in the total amino acid content remains constant after air curing, the proportion of free Gln and Glu decreases significantly. In contrast, the proportion of Asn and Asp increases dramatically after air curing, representing 33% of the total and 75% of the free amino acid content. This suggests that amino acid accumulation and protein degradation involving Asn, Asp, Glu, and Gln are differentially regulated during air curing.

During senescence, asparagine synthetase catalyzes the adenosine triphosphate (ATP)-dependent transfer of the amine group of Gln to Asp. This reaction generates Glu and Asn [30]. We hypothesized that asparagine synthetase participates in Asn formation during leaf curing, and we performed a time-course experiment to quantify Asn, Gln, Asp, and Glu contents during the air curing process (Figure 1b). We correlated the fluctuations in amino acid content with the changes in tobacco leaf color, which reflects the different stages of senescence during air curing (Figure 1b). At harvest, fully mature tobacco leaves are still green. Then, during the first 2–3 days of curing, the chlorophyll levels decrease rapidly, reflecting the senescence-triggered “yellowing phase” [31]. The first yellowing symptoms appear on the leaf tip and propagate slowly into the leaf blade, until the blade becomes completely yellow after about 10 days of curing. By this point, the chlorophylls are fully degraded, faster than carotenoids and anthocyanin. Hence, the tobacco leaf appears red-orange in color. In parallel, the leaf tissue dries out progressively and loses its water content to become completely brown-orange after 45 days of curing. We observed a substantial increase in Asn content—up to 36.8 mg/g dry weight (DW)—during the first 8 days of curing, which is then maintained until the end of curing. This suggests that Asn is only poorly degraded by asparaginases after the yellowing phase (10–12 days post-harvest), when the photosynthetic apparatus is degraded and not able to participate in the biosynthesis of new catabolites [31]. Interestingly, the Gln content increases transiently to reach a maximum of 15 mg/g DW 4 days after harvest, decreases rapidly and strongly after 5 and 15 days of curing, and remains very low until the end of curing. This suggests that Gln could contribute to the formation of Asn during the “yellowing phase” through the activity of asparagine synthetases (Figure S3). Although the average Asp and Glu levels remain low compared with Asn and Gln levels, we observed significant changes in their concentrations during the time course of air curing (Figure 1b). The Asp content is higher at harvest, then decreases by 75% during the first 4 days of curing, and increases again to remain between 2 and 3 mg/g DW. Glu levels increase transiently by 1.4-fold during the first 2 days and then remains very low until the end of curing. This suggests that Gln and Asp pools are rapidly and constantly used for Asn production during early curing, and that Glu is transiently produced and then quickly recycled within other biochemical pathways. Overall, these data indicate that Asn is the major free amino acid produced during curing, while Gln is possibly the amine donor and Asp the acceptor.

### 2.2. Asparagine Synthetases are Expressed Differently in Tobacco Organs and During Air Curing

**Figure 2:**
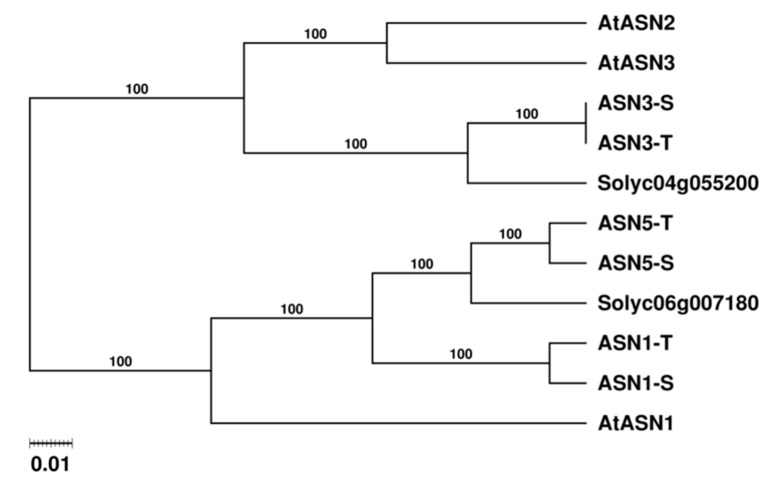
Molecular phylogenetic tree of plant asparagine synthetases (ASN). The ASN genes of *Nicotiana tabacum* originate from ancestors of *Nicotiana sylvestris* (S) or *Nicotiana tomentosiformis* (T). Tobacco polypeptides ASN1-S, ASN1-T, ASN3-S, ASN3-T, ASN5-S, and ASN5-T were compared with Arabidopsis proteins AtASN1 (At3g47340), AtASN2 (At5g65010), and AtASN3 (At5g10240) and tomato proteins Solyc06g007180 and Solyc04g055200. The tree was constructed by using the PHYLIP software, and the numbers are bootstrap values. The scale indicates the distance between two sequences, based on the distance matrix from MUSCLE alignment.

To determine whether the accumulation of Asn in tobacco leaf tissue during air curing is a result of the activity of asparagine synthetases, we characterized the complete expression profiles of some asparagine synthetase genes in tobacco under growing and air curing conditions. We first screened the tobacco genome to identify asparagine synthetase genes. Tobacco is an allotetraploid which has evolved through interspecific hybridization of *Nicotiana sylvestris* and *Nicotiana tomentosiformis.* We identified six full-length asparagine synthetase genes and their corresponding transcripts in Nicotiana tabacum. These genes originate from ancestors of *Nicotiana sylvestris* (S) or *Nicotiana tomentosiformis* (T). We named them *ASN1-S*, *ASN1-T*, *ASN3-S*, *ASN3-T*, *ASN5-S,* and *ASN5-T*. *Arabidopsis thaliana* has three full-length genes, namely *AtASN1*, *AtASN2* and *AtASN3* [32], and tomato (*Solanum lycopersicum*) has two full-length genes, *Solyc06g007180* and *Solyc04g055200* [33], related to asparagine synthetase. The results of phylogenetic analyses of the corresponding gene products are depicted in Figure 2, which shows two different groups of asparagine synthetases within the plant genome. The first group comprises *Solyc04g055200* and *AtASN2* and *AtASN3* which are close to *ASN3-S* and *ASN3-T* and belong to Dicot subclass II. The second group consists of *Solyc06g007180*, *AtASN1*, *ASN1-S*, *ASN1-T*, *ASN5-S*, and *ASN5-T*, which belong to Dicot subclass I [34]. Such a dichotomy means that these two groups might have different functions in the regulation of asparagine biosynthesis or might act in different tissues or cell compartments. The percentage of identity between proteins revealed that *ASN3-S* and *ASN3-T* are similar and very close to *Solyc04g055200* and *AtASN3* (85% identity; Table S2). This suggests a conservation of this gene cluster during evolution. *ASN1-S* and *ASN1-T* and *ASN5-S* and *ASN5-T* are homoeologs from *N. sylvestris* and *N. tomentosiformis* ancestors and intrinsically share a very high percentage of identity (>97%; Table S2). This indicates that the corresponding proteins might have a common ancestor with similar functions in planta. The common ancestral origin of tobacco chromosomes has been previously reported by using COSII markers [35], SSR markers [36], and genomic sequences [37]. Genetic mapping of *ASN* genes in the tobacco genome confirms the common ancestral origin of *ASN1* and *ASN5* genes. The *ASN5-S* genomic sequence is identified on tobacco chromosome 8, while *ASN5-T* is on tobacco chromosome 22. These two tobacco chromosomes share the same ancestor as tomato chromosome 6, where tomato asparagine synthetase 1 (*Solyc06g007180*) is mapped. The genomic sequence of *ASN1-S* is identified on tobacco chromosome 3, while that of *ASN1-T* is on tobacco chromosome 17. These two tobacco chromosomes share the same Solanaceae ancestor, which corresponds to tomato chromosome 2. Although there is no identified ASN gene on the tomato reference genome Chr2, some ancestral link might exist between tomato Chr2 and tomato Chr6 [35] which would suggest a common ancestor between *ASN1* and *ASN5*. Hence, these two gene families of tobacco likely originate from a unique ancestor of the asparagine synthetase gene mapped on chromosome 6 of the common Solanaceae ancestor.

**Figure 3:**
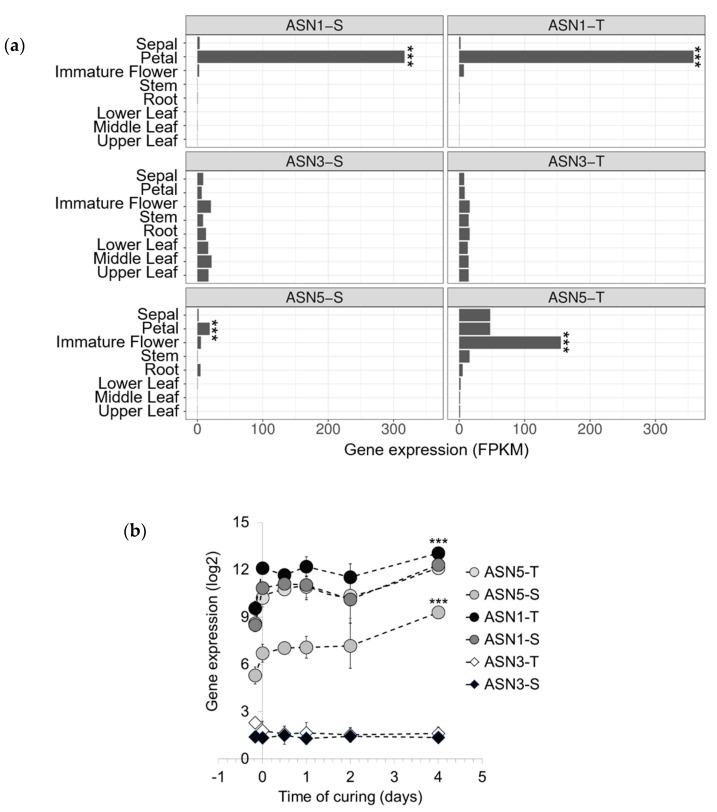
(**a**) Expression of asparagine synthetase (ASN) genes in different organs of burley tobacco. Tissue samples, including the sepal, petal, immature flower stem, root, and leaf in lower, middle, and upper positions, were collected from burley TN90 tobacco plants at flowering stage when lower leaves just started to senesce and became ready for curing, and were then used for RNA-seq analysis. Each bar represents the averaged FPKM (Fragments Per Kilobase of exon per Million fragments mapped) values corresponding the relative expression levels of the gene. Data were collected from three biological replicates and analyzed by *p* values adjusted for false discovery rate. Asterisks indicate statistical significance when comparing expression levels among tissues (n = 3, *** *p* < 0.001). (**b**) Time course of the air curing effect on the asparagine synthetase (ASN) gene expression in burley tobacco leaves. The expression patterns of *ASN* genes (*ASN1-S, ASN1-T, ASN3-S, ASN3-T, ASN5-S,* and *ASN5-T*) were followed at early stages of air curing (1 to 4 days post-harvest). Data were collected from three biological replicates. Each datapoint represents the mean of gene expression (in log2) estimated by using the Affymetrix Tobacco exon array in accordance with Martin et al. [39]. Vertical bars are standard deviation (SD).

We determined the spatial expression profiles of *ASN* genes in burley tobacco plants grown in the field and harvested at the flowering stage (Figure 3a). One characteristic feature of tobacco leaf senescence is that the leaves senesce gradually from the lower to the upper stalk [38]. Thus, the leaves at lower positions (i.e., source) which are the first to senesce, are a reservoir of carbon and nitrogen including amino acids, for the younger leaves, flowers, and seeds at the upper position (i.e., sink). We measured the transcript levels of *ASN* in the sepal, petal, immature flower, stem, root, and lower, middle, and upper leaves. Analysis of *ASN1-S* and *-T* and *ASN5 -S* and *-T* transcript levels revealed that *ASN5-T* is preferentially expressed in flowers (immature flower, petal, and sepal), whereas *ASN1-T* and *ASN1-S* are strongly expressed in petals. Similarly, *ASN5-S* is also expressed in petals, only to a much smaller degree but with similar statistical significance (q value < 0.001). Such high expression of *ASN1-S* and *-T* and *ASN5-S* and *-T* in flowers suggests that Asn is also actively produced in sink tissues. In contrast, *ASN3-S* and *ASN3-T* exhibit basal expression levels in all organs, suggesting that these genes are likely involved in the synthesis of a constitutive pool of Asn, at least at this stage of development. Additionally, the differences in the expression profiles of *ASN5-T/S*, *ASN1-T/S*, and *ASN3-T/S* correlate with the phylogenetic analyses, with *ASN3-T/S* clustering separately from the remaining four genes.

To evaluate the importance of *ASN* genes during air curing, we investigated their expression profiles in burley tobacco leaf tissue at early stages of curing. To this end, we plotted *ASN* gene expression data from Tobacco exon array Affymetrix gene chips ([39]; Figure 3b). The data showed that *ASN3-S* and *ASN3-T* are maintained at very low expression levels after harvest and during air curing. In contrast, *ASN1-S*, *ASN1-T*, and *ASN5-T* are already expressed at harvest, and their levels of expression increase significantly during curing (3200-fold induction of *ASN1-S*, 4100-fold induction of *ASN1-T*, and 4400-fold induction of *ASN5-T* after 4 days of curing, relative to the levels at the start of curing). Similarly, *ASN5-S* exhibits the same expression pattern, but with a lower magnitude (530-fold induction relative to the level at the start of curing). The strong expression of these genes suggests that asparagine synthetases are activated and can contribute to Asn production during air curing.

### 2.3. Different Air Curing Methods Significantly Impact the ASN Gene Expression and Aspartate, Glutamine, Glutamic Acid, and Asparagine Production

**Figure 4:**
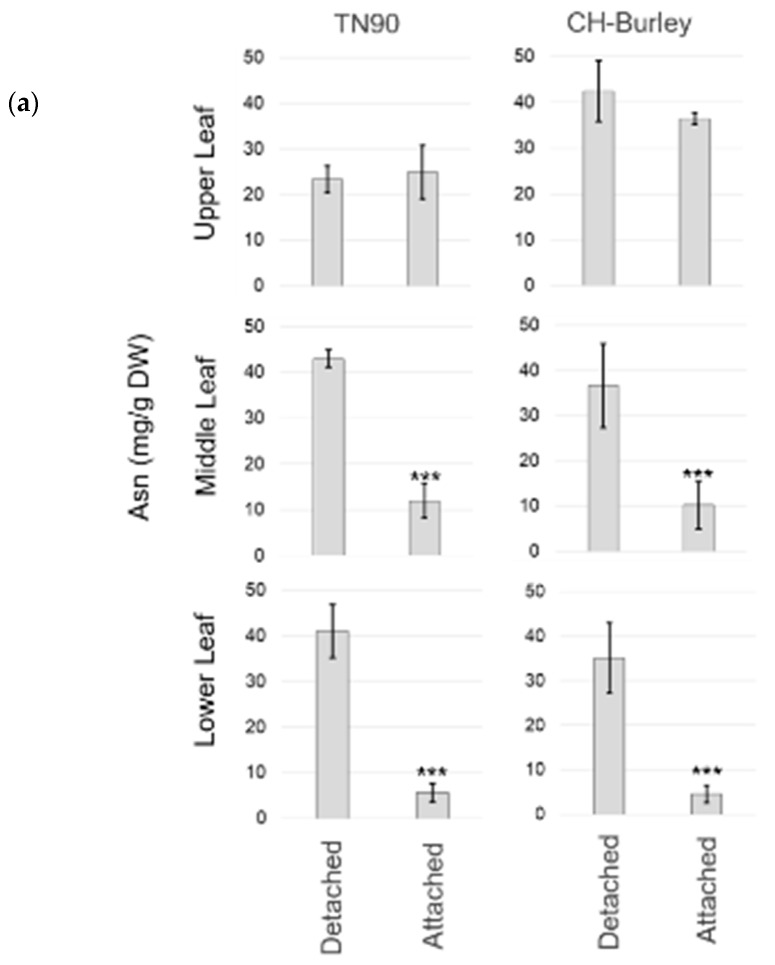

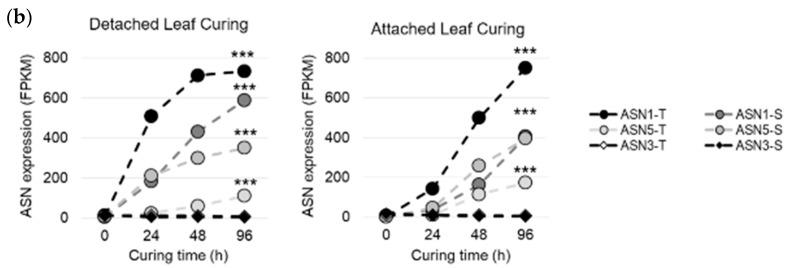
(**a**) Asparagine (Asn) content in air cured lower, middle, and upper leaves of two burley varieties (TN90 and CH-Burley). Leaf curing was performed either individually on string (Detached leaf) or on the stalk (Attached leaf) for 50 days. Data were collected from three biological replicates. The data are summarized in histogram bars, in which each bar represents the mean Asn content and the error bar represents the standard deviation (SD). Asterisks indicate statistical significance when comparing detached and attached leaf-curing conditions (n = 3; *** *p* value < 0.001; t-test). (**b**) ASN gene expression in the middle leaf position during the first 96 h of air curing either on string (Detached leaf curing; upper panel) or on stalk (Attached leaf curing; lower panel). Leaf samples from burley TN90 plants were collected during curing and used for RNA-seq analysis. Each data point is calculated from the averaged FPKM (Fragments Per Kilobase of exon per Million Fragments Mapped) values and represents the relative expression level of the gene. Data were collected from three biological replicates. Vertical bars are SD. Asterisks indicate statistical significance when comparing the expression levels at the start and after 4 days of curing (n = 3; *** *p* < 0.001; t-test).

Curing in barn is the method of choice for curing burley tobacco [40,41]. Usually, burley leaves are cured while still attached to their stalk (attached leaf) to dry out. In some cases, burley leaves can also be cured on string after being detached from their stalk (detached leaf). We evaluated the impact of stalk removal on Asn formation. We hypothesized that there would be differences in Asn accumulation between leaf curing (detached leaf) and stalk curing (attached leaf). We checked the impact of both curing processing methods on Asn accumulation in the leaves of two varieties of burley tobacco, TN90 and CH-Burley (Figure 4a). We analyzed the Asn content in cured leaves from the lower, middle, and upper positions of the stem. Both varieties of burley tobacco show similar Asn concentrations, which suggests that the mechanisms regulating Asn production seems to be well- preserved among the burley cultivars, although the concentration of Asn is higher (2-fold) in the upper leaves of CH-Burley. In contrast, there are large differences in Asn accumulation between cured detached and attached leaves, with the accumulation being substantially higher in lower and middle detached leaves (about 36–43 mg/g DW) than in cured attached leaves (<12 mg/g DW). This suggests either a decrease in the production of Asn, a degradation or a rapid remobilization of Asn in sink tissues during stalk curing compared to leaf curing, with the latter having individual leaves not connected to sink organs during the curing process. No significant differences were observed in upper leaves when comparing cured detached and attached leaves, which suggests that the mechanisms that regulate Asn accumulation in these sink tissues are different than those in lower and middle leaves, in which the yellowing phase occurs earlier than in upper leaves.

Therefore, we investigated whether the differences in Asn accumulation were linked to the activity of asparagine synthetases. We quantified the accumulation of *ASN* transcripts over time in middle leaves attached to their stalks during curing or detached from the stalk before curing (Figure 4b). Interestingly, we observed a rapid and strong upregulation of *ASN1-T* expression at the onset of curing in detached leaves (509 FPKM; 24 h post-curing). In comparison, the induction of this gene is much slower (143 FKPM; 24 h post-curing) in attached leaves. However, the same level of expression is eventually reached after 96 h in both curing processes. Similarly, *ASN1-S* and *ASN5-S* are induced rapidly at the onset of curing in detached leaves while being expressed only after 24 h of curing in attached leaves. In addition, the induction of *ASN1-S* is stronger in cured detached leaves than in cured attached leaves. No significant difference is observed between leaf and stalk curing in terms of *ASN3-T* and *ASN3-S* expression, with these two genes being not significantly induced during curing.

The induction of *ASN-1* and *ASN-5* transcripts during leaf curing is correlated with the expression of senescence markers such as *SAG12* and *RBCS* gene expression ([42,43]; Figure S4a,b). As expected, the expression of the senescence-associated gene *SAG12* increases, whereas that of the photosynthetic gene *RBCS* decreases over time (Figure S4a). In addition, abscisic acid (ABA), a hormonal compound produced during leaf senescence [44], was found to accumulate in both cured detached and attached leaves (Figure S4b).

**Figure 5:**
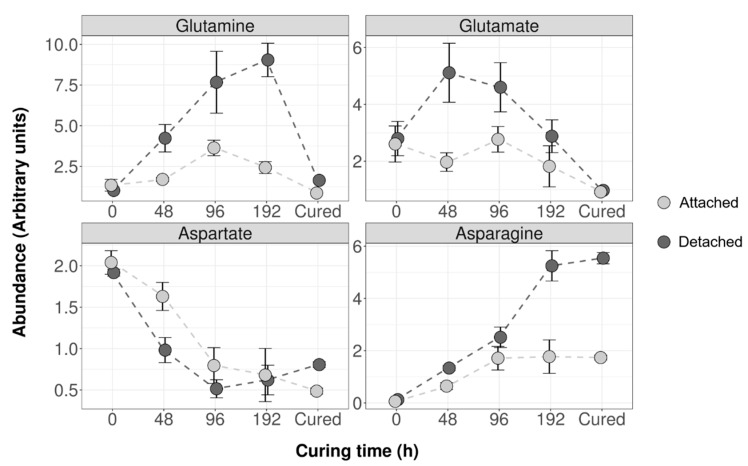
Asn, Gln, Asp, and Glu abundance during the curing time course in detached and attached leaves. Arbitrary units are used for quantification of each metabolite. Quantification data of Asn, Gln, Asp, and Glu are presented at harvest, after 48, 96, and 192 h of curing, and at the end of curing (Cured). Datapoints are the mean of three biological replicates (n = 3). Vertical bars are standard deviation (SD).

Finally, we compared the accumulation of Glu, Gln, Asp, and Asn between detached and attached leaves during the curing time course (Figure 5). The metabolomic data show 70% less Asn accumulated in cured attached leaves relative to the levels in detached leaves, which is consistent with our previous observations (Figure 4a). Interestingly, the major accumulation of Asn occurs after 4 days (96 h) of curing and is correlated with the increase in the levels of Gln, a potential donor for Asn synthesis (Figure S3). Although the Asp content is significantly higher in attached leaves than in detached leaves after 48 h of curing, we observed a decrease in Asp concentrations in both curing methods.

### 2.4. Silencing of ASN1 and ASN5 Affects the Production of Asp, Asn, and Glu

**Figure 6:**
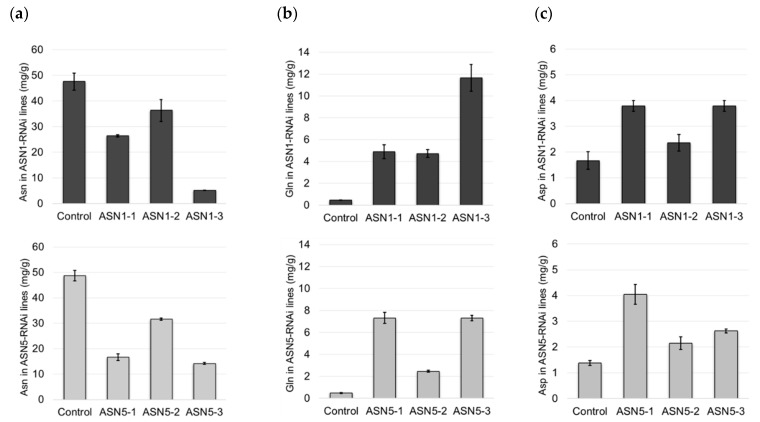
Asparagine, glutamine, and aspartate content in middle leaf lamina of wild-type, *ASN1-RNAi*, and *ASN5-RNAi* tobacco plants at the end of detached leaf curing. (**a**) Asparagine (Asn), (**b**) Glutamine (Gln), and (**c**) Aspartic acid (Asp) levels were measured in bulk leaves from 15 T1 transformed burley plants. Data were determined from experiments on three independent transformation events. The data are summarized in histogram bars, in which each bar signifies the amino acid content in mg/g dry weight, and the error bar represents the standard deviation (SD).

We used a genetic approach to validate the function of *ASN1* and *ASN5* gene families in Asn production during curing. We generated constructs to silence *ASN1 S/T* and *ASN5 S/T*, respectively. We designed a DNA stretch that was identical in both *ASN1 S/T* and *ASN5 S/T* for silencing (see Material and Methods and Figure S5). The RNAi constructs were expressed in burley tobacco under the control of the constitutive MMV (Mirabilis mosaic virus) promoter. For each construct, we performed qPCR experiments to check the silencing of *ASN* genes in three independent T1 transformants, designated *ASN1-1*, *ASN1-2*, and *ASN1-3* for *ASN1-RNAi* lines and *ASN5-1*, *ASN5-2*, and *ASN5-3* for *ASN5-RNAi* lines (Figure S6a). Relative to the wild-type plants (control), the *ASN1-* and *ASN5-RNAi* lines exhibit a strong and significant downregulation of *ASN1* and *ASN5* genes, respectively, during air curing. Leaf biomass and nicotine content is not impacted by the silencing of *ASN1* and *ASN5* genes (Figure S6b,c), indicating that these genes have no or very little impact on plant growth and nicotine synthesis in our experimental settings. This implies that ASN1 and ASN5 leaf activities are completely restricted to post-harvest maturation and disconnected from plant growth and nicotine pathways. We measured the amino acid content in bulk cured leaves (middle stalk position) collected from 10 *ASN-RNAi* plants of each line at the end of air curing (Figure 6). The Asn concentration is significantly lower in all *ASN1-* and *ASN5-RNAi* lines than in the wild-type plants. This result confirms that Asn is produced during curing and validates the importance of ASN1 and ASN5 in the regulation of Asn synthesis. Similarly, we quantified Gln and Asp levels at the end of curing. Both *ASN1-* and *ASN5-RNAi* lines exhibit a hyperaccumulation of these amino acids relative to the wild-type plants, indicating that ASN1 and ASN5 are critical for regulating Asn synthesis in cured tobacco leaves.

## 3. Discussion

Detached tobacco burley leaves show a dramatic and rapid accumulation of Asn after harvest. Asn has long been described as an important form of nitrogen reallocated from source senescing tissues to sink developing organs. Our data indicate that changes in Asn content are correlated with the onset of senescence that occurs during curing.

We hypothesized that the strong increase in Asn content in leaves detached from their stalk results from either degradation of proteins by proteases or de novo synthesis. However, this accumulation of Asn seems to result not directly or solely from proteolytic activities but from active synthesis. Importantly, this accumulation of Asn persists even when photosynthesis is shut down. Such dark-induced senescent activities have been observed in other plant species, including sunflower [45], *Arabidopsis* [14], and barley [46]. Although this needs to be confirmed by labelling experiments, the rapid increase and decrease in Asp and Gln levels early during leaf curing suggest that both Asp and Gln pools are involved in Asn synthesis. To this end, the amino acid group of Gln is transferred to Asp through the activity of asparagine synthetases. This transfer requires energy provided by adenosine triphosphate (ATP). It is possible that ATP production results from the activity of the photosynthetic apparatus, still effective at the beginning of curing. However, with chloroplasts being degraded rapidly during senescence, the source of ATP involved in this reaction is unlikely to come from photosynthesis at later stages of curing [47]. Early works on senescence have shown a concomitant increase in ethylene and ATP content during leaf senescence [48]. More recently, an increase in ATP/ADP ratio has been reported in senescent leaves of autumnal trees, in which chloroplasts are fully degraded [49]. It has been suggested that ATP might originate from the activity of mitochondria during senescence of leaf tissues [49]. Another study has reported that *Arabidopsis* plants under dark-induced senescence show a rapid decline in photosynthetic capacity, while maintaining high mitochondrial respiration and ATP production [50]. Similarly, the production of ATP for Asn synthesis during tobacco curing might result from mitochondrial respiration. Further research needs to be conducted to validate this hypothesis. The catalytic reaction that generates Asn also produces Glu. Although we would expect a rise in Glu content during leaf curing, the basal level of Glu remains quite low. During nitrogen assimilation, Glu usually serves as an ammonium donor for the synthesis of many other amino acids, including Asn [51]. It is likely that Glu also contributes to Asn production during leaf senescence; its reconversion to Asn or other amino acids would, hence, explain its constant low basal levels during curing. However, we cannot exclude the involvement of Glu in other biochemical pathways possibly still active during the curing process, as observed previously during leaf senescence [52,53].

The biosynthesis of Asn during curing is catalyzed by specific asparagine synthetases. In the present study, four tobacco gene products—*ASN1-S*, *ASN1-T*, *ASN5-S,* and *ASN5-T*—were shown to be clustered with *AtASN1* and the tomato orthologous gene *Solyc06g007180*. *ASN1* and *ASN5* catalyze Asn production during leaf senescence. Our data suggest that *ASN1* and *ASN5* genes are the major contributors to Asn synthesis during curing. The constitutive expression of *ASN1-S* and *-T* genes in flowers also suggests that ASN1 participates in nitrogen re-assimilation in these organs to ensure nitrogen storage in seeds. In *Arabidopsis*, similar expression patterns associated with an increase in the total protein content in sink tissues were observed when overexpressing *AtASN1* [54]. This suggests that the group comprising *AtASN1*, *Solyc06g007180*, *ASN1,* and *ASN5* is devoted to the translocation of nitrogen from source leaves to seeds, thus allowing proper seed germination and better tolerance of seedlings to nitrogen-limiting conditions.

Silencing *ASN1* or *ASN5* tobacco gene families caused a strong reduction in Asn levels in cured tobacco leaves and confirmed the importance of these genes for the metabolism of nitrogen during curing. Interestingly, plant biomass, vegetative growth, and flowering time are not affected by this silencing. This indicates that nitrogen regulation in vegetative tissues, including Asn production, is not affected by the knockdown of *ASN1-S/-T* and *ASN5-S/-T*. In comparison, the presence of these genes in other crops like potato is fundamental to an efficient Asn production [55]. Consequently, subtle strategies are required to reduce Asn concentrations and, thus, acrylamide content in planta. By comparing different species, we noticed that several diploid species, including *Zea mays*, *Brassica oleracea*, *Sandersonia aurantiaca*, *Triphysaria versicolor*, *Solanum lycopersicum,* and *Arabidopsis thaliana*, have only one *ASN* gene copy close to *AtASN1*, whereas diploid *Nicotiana* species such as *N. tomentosiformis*, *N. sylvestris*, and *N. glauca* harbor two copies (unpublished data). This suggests that the *Nicotiana* genus acquired two gene copies (*ASN1* and *ASN5*) during evolution, possibly to enhance nitrogen metabolism and re-assimilation in flowers and senescing leaves. Our results indicate that the tobacco *ASN3* copies do not contribute to Asn accumulation during air curing. In *Arabidopsis*, *AtASN2* and possibly *AtASN3* localize in the phloem and appear to be essential for nitrogen assimilation, distribution, and remobilization in green tissues [38,56]. The results of amino acid sequence analysis revealed that *ASN3-S* and *ASN3-T* as well as *Solyc04g055200* and *AtASN3* are phylogenetically close. This suggests that the tobacco *ASN3-S* and -*T* might also contribute to Asn accumulation within vegetative tissues, but not during curing.

Interestingly, the leaf and stalk curing methods present significant differences in the onset and temporal activity of asparagine synthetases as well as accumulation of Asn. The rapid induction of *ASN-1* and *ASN-5* transcripts in cured detached leaves relative to that in cured attached leaves is correlated with a rapid decrease in *RBCS* expression level and induction of *SAG12*. In addition, abscisic acid (ABA) accumulates more rapidly in detached leaves than in attached leaves during curing. This might be due to the fact that dehydration occurs quickly in detached leaves compared to leaves attached to their stalk. Our results suggest that the senescence process is strongly induced when leaves are detached from their stalk, molecular exchanges between the stalk and leaves potentially delaying the onset of senescence in stalk leaves. Although we observed a decrease in Asp concentrations in both curing methods, which is consistent with our hypothesis that Asp acts as an amine acceptor, the Asn content is lower in cured attached leaves than in detached leaves.

We hypothesize that, because it cannot be transported to sink organs, Asn accumulates in excess in detached senescing leaves during curing. In contrast, the absence of Asn accumulation in source leaves of tobacco attached to their stalk suggests rapid remobilization of nitrogen during air curing. One factor that can explain these differences is the quality and quantity of light. Leaves or stalks are left in the dark or in dim light during air curing. Previous studies have described that dark-stress conditions induce senescence in individual leaves while delaying the onset of senescence in whole plants such as *Arabidopsis* and sunflower [57,58]. Although some photoreceptors have been shown to regulate senescence, the underlying molecular mechanisms that link light perception and signaling and Asn production are yet to be described. Nitrogen re-assimilation and remobilization often depends on nutritional and environmental conditions and usually occurs in plants well-fertilized with nitrate [46], which is the case for burley tobacco varieties relative to other tobacco cultivars like Virginia tobacco. In barley, asparagine synthetase genes *HvASN* are repressed under low-nitrate conditions, while being induced by leaf senescence under high-nitrate conditions [46]. In contrast, other studies in sunflower have shown enhanced nitrogen remobilization in plants under low-nitrogen conditions relative to that in plants supplied with nitrogen [59]. Therefore, it should be considered that the responses observed in our experimental settings might not reflect those happening in the field or those occurring under nitrate-limiting conditions. Overall, this might indicate that different methods of curing can modify the Asn content in cured leaves and, consequently, the production of acrylamide in smoke or tobacco heated products.

## 4. Materials and Methods 

### 4.1. Tobacco, Field Growth and Curing Conditions

Burley tobacco cultivars TN90 and CH-Burley were grown in the field of La Sota (Payerne, Switzerland) for about 12 weeks by following common agricultural practices [60]. At harvest, burley tobacco leaves were air cured by hanging (attached or detached leaf from the stalk) in aerated barns in dim light, at temperatures between 10 ℃ and 35 ℃, and humidity of 40–80% for about 45 days. The exact number of days is reported in the legend of each experiment. During the first 10 days of curing, the leaf tissue dries out progressively and loses water, and chlorophyll is degraded. This phase is commonly termed the “yellowing phase”. Gradually, the leaf tissue keeps drying and becomes completely brown-orange after 45 days of curing.

### 4.2. ASN, SAG12, and RBCS Genes

ASN5-S (Nitab17:1830755-1837959, PMI082252-1.1), ASN5-T (Nitab17:26411492-26417838, PMI083123-1.1), ASN1-S (Nitab08:69530005-69534905, PMI035750-1.1), ASN1-T (Nitab0U:136382762-136384728, PMI054207-1.1), ASN3-S (Nitab11:34949226-34971482, PMI061708-1.1), ASN3-T (Nitab13:110192042-110209752, PMI070797-1.1, [39]). SAG12-cysteine proteinase (Nitab03:135546966-135548976, PMI012253-1.1), RBCS- RubisCO small subunit (Nitab21:84090081-84094562, PMI103582-1.1).

### 4.3. Generation of ASN1-RNAi and ASN5-RNAi Tobacco Plants

The specific DNA fragments selected for silencing both copies of *ASN1* (*ASN1-S* and *ASN1-T*) tcacatatattccctcataacacacccattacaaaggaagcatactactataggatgattttcgagcgctttttcccacagaattcagctgggctaaccgttcctggaggagcaagtgtggcgtgtagcacagctaaagctgtagagtgggatgcttcttggtcaaagaaccttgatccttcaggcagggctgctattggtgtacataactcggcttatgagaatcatgtacctgctatggctaatgggaatttgaccaaaaaaatcattggtcgtgtgccttctatggtagaagttggtgctgctcccgagctcacaataaagagttag and both copies of *ASN5* (*ASN5-S* and *ASN5-T*) gaccagattggagtgggatatatcaacatggtgatttttacttagcacatcaacgtttagcaattatcgatcctacttctggtgatcagcctctgtttaatcaagataagactattgttgttacagtcaatggagaaatttacaatcatgagaaacttcgtaatcttatgcctaatcacaagttcagaaccggaagtgattgtgatgttattgcacatctttatgaagaatatggagaaaattttgtggacatgttggatggggtgttctcttttgtattgttggatacgcgcgataacagctttcttgctgctcgtgatgcgattggaattactcccctctatattggttggggacttgatgg were cloned between the strong constitutive MMV promoter and the 3′ nos terminator sequence of the nopaline synthase gene of *Agrobacterium tumefaciens* [61]. The burley tobacco line TN90e4e5e10 (Zyvert TM) was transformed with each of these two constructs by using standard *Agrobacterium*-mediated transformation protocols [62]. TN90e4e5e10 (Zyvert) represents a selection from an ethylmethane sulfonate-mutagenized burley population that contains knockout mutations in CYP82E4, CYP82E5v2, and CYP82E10 [63] to prevent nornicotine production. The use of this line can avoid potential complications when interpreting future tobacco-specific nitrosamines (TSNA) data. Ten independent T0 plants from each construct were assayed by qPCR to select transgenic lines. Seeds were harvested from three T0 lines exhibiting strong *ASN* gene silencing. To verify that the T1 progeny inherited the RNAi gene fragment and displayed efficient gene silencing, RNA was isolated from 4 transgenic plants (out of 15) of each independent transformation event and their corresponding control plants, and qPCR experiments were performed to assess *ASN* gene expression levels.

### 4.4. Analysis of Total and Free Amino Acids 

Free amino acids were analyzed by SGS-MScan (Geneva, Switzerland) in accordance with the method described by Moldoveanu [64]. Total amino acid analysis was performed by conventional acid hydrolysis with 6 M hydrochloric acid (HCl) for 24 h at 110 °C, followed by derivation with phenylisothiocyanate (PITC)—except for proline, which was derivatized with 9-fluorenylmethylchloroformate (FMOC-Cl)—and loading onto High Performance Liquid Chromatography (HPLC) columns (Agilent 1200 HPLC system for automated derivation of the liberated amino acids).

### 4.5. Analysis of Amino Acids, Nicotine, and Nitrates by HPLC-MS

Four mature leaves at the mid-bottom position were collected from each plant and subjected to air curing for 7 weeks. The concentrations of amino acids and nicotine in the cured leaf lamina were determined by UHPLC-MS (Dionex Ultimate 3000). Samples of ground tobacco (~30 mg) were extracted with ethanol–water (1:1; 6 mL) for 45 min at 50 °C. The centrifuged extracts were diluted 10-fold. Liquid chromatographic separation was performed on an amide column (Waters Acquity BEH amide, 2.1- x 150-mm; 1.7 µm) at 45 °C; elution was performed with a gradient of 2 mM ammonium formate in water + 0.25% formic acid (eluent A) and acetonitrile + 0.1% formic acid (eluent B) by applying an elution gradient (0 min – 10% A, 0.5 min – 10% A, 4 min – 60% A, 4.5 min – 60% A, 4.6 min – 10% A, 6.8 min – 10% A; flow rate, 0.5 mL/min). For mass spectrometric detection of amino acids, a Q Exactive instrument (Thermo Scientific, Basel, Switzerland) was used in positive-ion electrospray ionization (ESI) mode for acquiring full-scan mass spectra. Nicotine concentrations in the extracts were calculated by using the peak-area integration method (peak in the 260-nm trace of a photodiode UV-VIS detector).

### 4.6. Metabolomic Analysis: Dipeptides, Asn, Gln, Asp, Glu, and Abscisate Measurement

Sample preparation and data acquisition were acquired by Metabolon (Metabolon, NC, USA). In brief, leaf samples were lyophilized, ground, and extracted in methanol. The resulting extract was divided into five fractions: two for analysis by two separate reverse-phase (RP)/UPLC-MS/MS methods by using positive-ion ESI mode, one for analysis by RP/UPLC-MS/MS by using negative-ion ESI mode, one for analysis by HILIC/UPLC-MS/MS by using negative-ion ESI mode, and one reserved for backup. The samples were placed briefly on a TurboVap® (Zymark) to remove the organic solvent. Raw data were extracted, peak-identified, and quality control (QC) processed by using Metabolon’s hardware and software. Metabolite quantitation was performed by peak area integration. The QC metrics for these study data and a summary of the total number of biochemicals detected are reported. Several types of QC samples were analyzed, including: 1) technical replicate samples derived from a pool of well-characterized human plasma (MTRX) or, alternatively, generated by combining a small portion of each (non-plasma) experimental sample (CMTRX), spaced evenly among the experimental samples; 2) extracted water samples (process blanks) and solvent blanks; and 3) a cocktail of QC standards, carefully chosen not to interfere with the measurement of endogenous compounds, spiked into every analyzed sample, allowing monitoring of instrument performance and aiding chromatographic alignment.

### 4.7. qPCR Experiments

Total RNA was isolated from tobacco leaf tissues by using the RNeasy Plant Mini Kit (Qiagen, Hilden, Germany). The RNA was digested by using RQ1 RNase-free DNase (Promega, Madison, WI, USA), and the DNase reaction was stopped by using the RQ1 DNase stop solution (Promega, Madison, WI, USA). The RNA was reverse-transcribed by using an oligo dT15 primer, dTNPs, RNasin Plus RNase inhibitor, and M-MLV reverse transcriptase, RNase (H-), Point Mutant (all from Promega, Madison, WI, USA). qRT-PCR was performed by using the Mx3005P system (Stratagene, Agilent, Waldbronn, Germany). Amplification reactions were performed by using ABsolute Blue SYBR Low Rox Mix (Thermo Scientific, Surrey, UK). For each target, different primer pairs were designed and tested for primer dimer formation and performance in a first qPCR run. Their efficiency was tested by plotting a standard curve with a five-fold dilution of cDNA. The *ACTIN9* gene was used as the internal standard. The following qPCR primers were used in this study: *ASN1*-forward, tcacatatattccctcataacacac; *ASN1*-reverse, aagaagcatcccactctacagctt; *ASN5*-forward, atatcttccctcacaacactccaact; *ASN5*-reverse, ctaccggaaggatcaaggttgt.

### 4.8. RNA-seq and Gene Expression Analysis from Affymetrix Tobacco Exon Array

Total RNA was isolated from tissue samples (triplicates, n = 3) by using the RNeasy mini kit (Qiagen, Hilden, Germany). Before cDNA library construction, RNA purity and integrity were checked by using Nanodrop (Qiagen, Hilden, Germany). mRNA was enriched from total RNA by using polyA to generate cDNA libraries for sequencing. All libraries were sequenced on an Illumina HiSeq-2500 sequencer by using version 3 chemistry and flow cells with runs of 2 × 100 bases. Sequence reads were mapped to the genomes by using Hisat2 (v. 2.1.0) [65]. Previously published gene models were used as the basis for differential gene expression analysis [39]. Gene expression changes were calculated using Cuffdiff2 (v. 2.2.1) based on previously published gene models [66]. Differential expression was considered significant if the associated FDR-corrected significance value provided by the software was below the 0.05 threshold. Tobacco proteins were then identified by performing a BLAST search against a database of transcripts (e-value cutoff, 1e-80).

Affymetrix Tobacco exon array is a whole genome exon array available for research use through Affymetrix array catalogue [39]. We retrieved information about *ASN* gene expression from the Gene Expression Omnibus (GEO) accession viewers GSE42319 [67] and GPL16290 [68] as indicated in Martin et al., 2012 [39].

### 4.9. Statistical Analyses

Our study employed different types of statistical analyses: (i) t-test with one-tailed distribution and two-sample equal variance; (ii) Welch’s two-sample t-test to test whether two unknown means are different in two independent populations; (iii) *p* values: the lower the *p* value, the greater the evidence that the null hypothesis (typically that two population means are equal) is not true; and (iv) q values: the false discovery rate (FDR) for a given set of compounds can be estimated by using the q value [69]. The q-value gives the FDR for a selected list (i.e., an estimate of the proportion of false discoveries for a list of compounds whose *p* values are below the cutoff for significance).

## 5. Patents

WO 2017/042162 Al; United States Patent Application 20190169628; Plants with reduced asparagine content.

## Supplementary Materials

The following are available online at https://www.mdpi.com/2223-7747/8/11/492/s1, Figure S1 (**a**): Nitrate and total alkaloid contents (% of dry weight [DW]) at harvest and after air curing (45 days post-harvest), Figure S1 (**b**): Total and free amino acid contents (mg/g of dry weight [DW]) at harvest and after air curing, Table S1: Distribution of total and free amino acid (%) contents in mature leaves of burley tobacco at harvest and after aircuring, Table S2: Number of senescence-activated protease genes significantly induced after 48 h of leaf curing, Figure S2: Dipeptide metabolite abundance during the curing time course, Figure S3: Asparagine (Asn) synthesis, Table S3: Percentage of identical residues in ungapped alignment regions between tobacco, tomato, and arabidopsis asparagine synthetase (ASN), Figure S4 (**a**): Expression of the senescence-marker gene *SAG12* and the small subunit of the Rubisco (*RBCS*) during the first 96 h of leaf curing on string (detached) or stalk (attached), Figure S4 (**b**): Variation of abscisate content during the first 96 h of air curing on detached or attached leaf, Figure S5 (**a**): *ASN1* and *ASN5* transcript levels in burley tobacco leaves, Figure S5 (**b**): Biomass of tobacco leaf tissues during growth, Figure S5 (**c**): Nicotine content in tobacco leaf tissues.
