# Supplementary material for: Asparagine Synthesis during Tobacco Leaf Curing
Source: Plants (Basel). 2019 Nov 11;8(11):492. doi: 10.3390/plants8110492 (PMC6918383; doi:10.3390/plants8110492)
Supplement: Supplementary file 1 [file plants-08-00492-s001.pdf]

plants

MDPI

Supplementary:

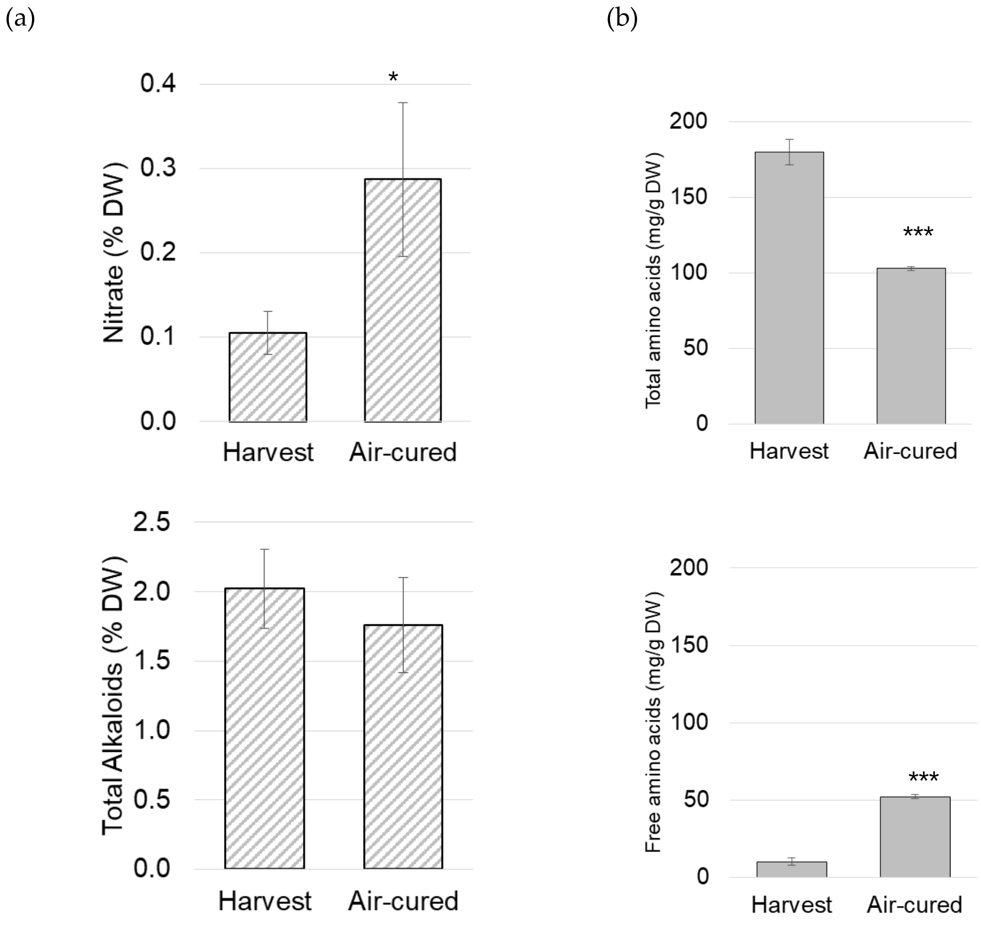

Figure S1. **(a)** Nitrate and total alkaloid content (% of dry weight [DW]) at harvest and after air curing (45 days post-harvest). Data were collected from three biological replicates. The average data are summarised in histograms, with error bars representing the SD. **(b)** Total and free amino acid content (mg/g of dry weight [DW]) at harvest and after air curing. The average data are summarised in histograms, with error bars representing the SD. Asterisks indicate statistical significance when comparing harvest to air cured conditions (Welch's two-sample t-test; n = 3; p *** < 0.001; * < 0.05).

Table S1. Distribution of total and free amino acid (%) content in mature leaves of burley tobacco at harvest and after air curing.

	**Total aa**		**Free aa**	
	**Harvested**	**Air Cured**	**Harvested**	**Air Cured**
Asp + Asn	11.4	32.6	24.2	76.1
Glu + Gln	14.1	10.4	31.9	4.5
Ser	4.9	5.1	3.3	3.0
His	2.5	2.6	2.8	2.2
Gly	6.0	4.6	0.4	0.6
Thr	5.3	3.8	5.5	0.9
Arg	6.3	4.8	1.2	1.2
Ala	6.6	5.0	2.7	1.2
Tyr	3.9	2.0	1.4	0.1
Val	5.5	3.7	2.1	0.4
Met	1.7	1.1	0.3	0.0
Trp	0.0	0.0	7.7	3.3
Phe	5.9	6.7	3.1	3.8
IIE	4.8	3.0	1.2	0.1
Leu	9.2	5.7	1.0	0.2
Lys	6.9	4.4	1.8	0.5
Pro	5.3	4.5	9.6	1.7
		100%		

Table S2. Number of senescence-activated protease genes significantly induced after 48 h of leaf curing. The transcript accumulation of known proteases was compared between harvest time and after 48 h of curing. The numbers of proteases significantly upregulated after 48 h curing are listed according to category (n = 3; $p < 0.05$).

Protease Coding Gene Families	
Alpha/beta-hydrolases superfamily protein	1
Aspartic proteinase A1 (APA1)	5
CLP proteas/crotonase family protein	2
Cysteine proteinases superfamily protein	10
DegP protease 3	0
Eukaryotic aspartyl protease family protein	9
FTSH protease 8	0
Gamma-glutamyl transpeptidase 4	1
Heat shock protein 101	2
Ion protease 1 & 3	0
Metallopeptidase M24 family protein	0
Papain family cysteine protease	1
Peptidase M20/M25/M40 family protein	1
Protease-related	1
SAG 12	1
Serine carboxypeptidase-like	4
SERPIN	2
Signal peptide peptidase	1
SITE-1 protease	1
Subtilisin-like ser endopeptidase fam prot.	5
Ubiquitin-specific proteases	3
Total	50

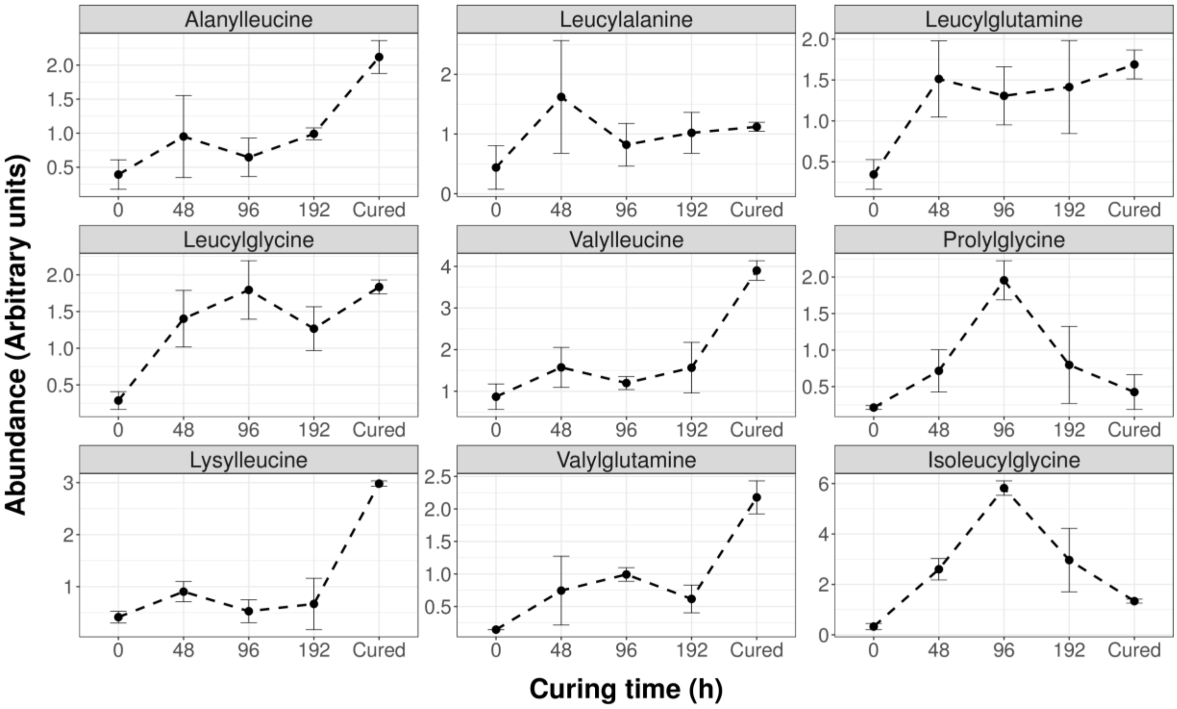

Figure S2. Dipeptide metabolite abundance during the curing time course. Arbitrary units are used for quantification of each metabolite. The abundance of alanylleucine, leucylalanine, leucylglutamine, leucylglycine, valylleucine, prolylglycine, lysylleucine, calylglutamine, and isoleucylglycine is

presented at harvest, after 48, 96, and 192 h of curing, and at the end of curing (Cured). Datapoints are the mean of three biological replicates (n = 3). Vertical bars are standard deviation (SD).

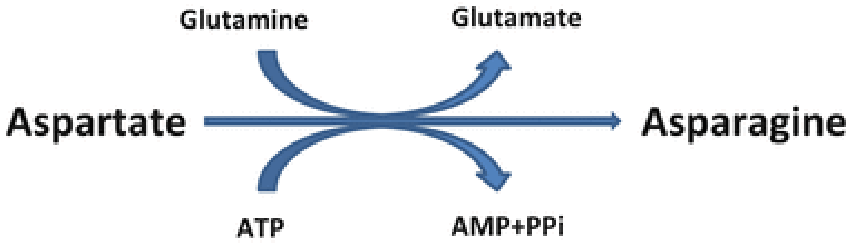

Figure S3. Asparagine (Asn) synthesis. The asparagine synthesis pathway involves adenosine triphosphate (ATP)-dependent transfer of the amino group of glutamine to aspartate by asparagine synthetase (ASN). This catalytic reaction generates glutamate and asparagine [24].

Table S3. **(a)** Percentage of identical residues in ungapped alignment regions between tobacco, tomato, and arabidopsis asparagine synthetase (ASN) gene products. (**b**) Percentage nucleotide sequence identity between *Nicotiana tabacum* asparagine genes.

(**a**)

	**AtASN1**	**AtASN2**	**AtASN3**	**NtASN5-T**	**NtASN5-S**	**NtASN1-T**	**NtASN1-S**	**NtASN3-T**	**NtASN3-S**	**Solyc06g007180**	**Solyc04g055200**
**AtASN1**	**100**	76.91	77.26	83.01	83.73	85.1	85.27	76.17	76.17	83.9	77.44
**AtASN2**	76.91	**100**	91.33	77.22	76.99	78.03	78.03	84.64	84.64	76.82	85.46
**AtASN3**	77.26	91.33	**100**	77.4	76.99	78.37	78.37	85.14	85.14	76.99	85.96
**NtASN5-T**	83.01	77.22	77.4	**100**	97.88	90.27	89.91	77.16	77.16	93.81	80.2
**NtASN5-S**	83.73	76.99	76.99	97.88	**100**	90.85	90.51	77.28	77.28	95.08	79.7
**NtASN1-T**	85.1	78.03	78.37	90.27	90.85	**100**	98.31	77.62	77.62	90.34	80.45
**NtASN1-S**	85.27	78.03	78.37	89.91	90.51	98.31	**100**	77.45	77.45	89.83	80.2
**NtASN3-T**	76.17	84.64	85.14	77.16	77.28	77.62	77.45	**100**	100	75.93	64.73
**NtASN3-S**	76.17	84.64	85.14	77.16	77.28	77.62	77.45	100	**100**	75.93	64.73
**Solyc06g007180**	83.9	76.82	76.99	93.81	95.08	90.34	89.83	75.93	75.93	**100**	79.95
**Solyc04g055200**	77.44	85.46	85.96	80.2	79.7	80.45	80.2	64.73	64.73	79.95	**100**

(**b**)

	**NtASN5-T**	**NtASN5-S**	**NtASN1-T**	**NtASN1-S**	**NtASN3-T**	**NtASN3-S**
**NtASN5-T**	**100**	96, 88	87, 1	86, 93	71, 31	71, 25
**NtASN5-S**	96, 88	**100**	87, 65	87, 48	71, 34	71, 28
**NtASN1-T**	87, 1	87, 65	**100**	97, 74	71, 86	71, 91
**NtASN1-S**	86, 93	87, 48	97, 74	**100**	72, 14	72, 2
**NtASN3-T**	71, 31	71, 34	71, 86	72, 14	**100**	99, 77
**NtASN3-S**	71, 25	71, 28	71, 91	72, 2	99, 77	**100**

(**a**)

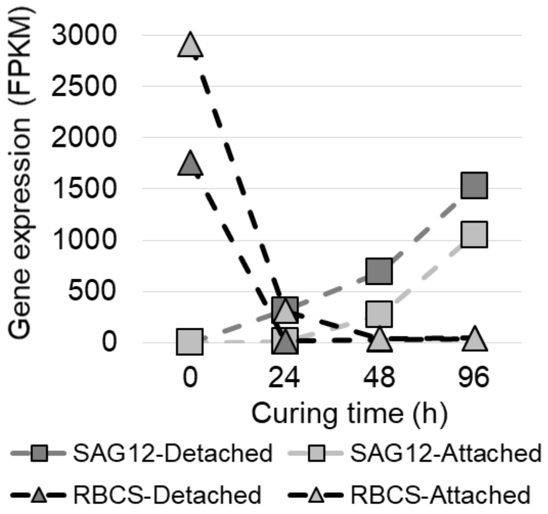

(**b**)

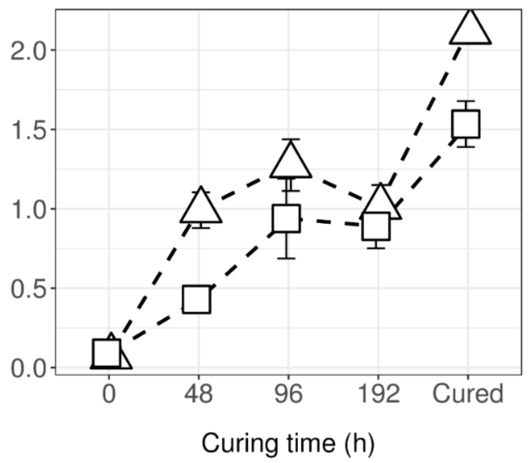

Figure 4. (a) Expression of the senescence marker gene *SAG12* and the small subunit of the Rubisco (*RBCS*) during the first 96 h of leaf curing on string (detached leaf) or stalk (attached leaf). Gene expression levels were quantified at different time points during air curing (h = curing time in hours). Data were collected from three biological replicates. Each datapoint represents the averaged FPKM (Fragments Per Kilobase of exon per Million mapped reads) values, which indicate the relative expression levels of the gene. Vertical bars signify the standard deviation (SD). Asterisks indicate statistical significance when comparing gene expression levels between the stalk- and leaf-curing methods (n = 3; ***$p < 0.0011$ t-test). **(b)** Variation in abscisic acid content during the first 96 h of air curing of detached or attached leaves. Data were collected from three biological replicates. Each datapoint represents the average abundance of abscisic acid in an arbitrary unit. Vertical bars are SD.

(a)

```
ASN1-S          ATGTGCGGGATCTTGGCTGTTTTGGGTTGTTCTGATGATTCTCAGGCCAAAAGGGTTCGT
ASN1-T          ATGTGCGGGATCTTGGCTGTTTTGGGTTGTTCTGATGATTCTCAGGCCAAAAGGGTTCGT
ASN5-T          ATGTGTGGGATCTTGGCTTTGTTGGGTTGTCCAGATGATTCTCAAGCCAAAAGGGTTCGA
ASN5-S          ATGTGTGGAATCTTGGCTTTGTTGGGTTGTTCAGATGATTCTCAGGCCAAAAGGGTTCGA
                ***** ** ********* * ********* * *********** **************

ASN1-S          GTTCTCGAGCTCTCTCGCAGGTTGAAGCATCGTGGACCAGATTGGAGTGGGCTGTATCAA
ASN1-T          GTTCTCGAGCTCTCTCGCAGGTTGAAGCATCGTGGACCAGATTGGAGTGGGCTGTATCAA
ASN5-T          GTTCTTGAGCTCTCTCGCAGGTTGAAGCATCGTGGACCAGATTGGAGTGGGATATATCAA
ASN5-S          GTTCTTGAGCTCTCTCGCAGGTTGAAGCATCGTGGACCAGATTGGAGTGGGATATATCAA
                ***** ********************************************* * ******

ASN1-S          CATGGGGACTGTTACTTGGCACATCAGCGTCTAGCTATTGTTGATCCTGCTTCCGGTGAT
ASN1-T          CATGGGGACTGTTACTTGGCACATCAGCGTCTAGCTATTGTTGATCCTGCTTCCGGTGAT
ASN5-T          CATGGTGATTTTTACTTAGCACATCAACGTTTAGCAATTATCGATCCTACTTCTGGTGAT
ASN5-S          CATGGTGATTTTTACTTAGCACATCAACGTTTAGCAATTATCGATCCTGCTTCTGGTGAT
                ***** ** * ****** ******** *** **** *** * ****** **** ******

ASN1-S          CAACCTCTGTTTAACGAAGATAAGACGATTGTTGTTACGGTAAATGGAGAGATCTACAAT
ASN1-T          CAACCTCTGTTTAACGAAGATAAGACGATTGTTGTTACG---------------------
ASN5-T          CAGCCTCTGTTTAATCAAGATAAGACGATTGTTGTTACAGTCAATGGAGAAATTTACAAT
ASN5-S          CAGCCTCTGTTTAATCAAGATAAGACGATTGTTGTTACAGTCAATGGAGAGATTTACAAT
                ** ***********  **********************

ASN1-S          CACGAGCAACTTCGTAAGCAAATGCCTAATCATAAGTTCCGGACTGGCAGTGACTGTGAT
ASN1-T          ------------------------------------------------------------
ASN5-T          CATGAGAAACTTCGTAATCTTATGCCTAATCACAAGTTCAGAACCGGAAGTGATTGTGAT
ASN5-S          CATGAGAAACTTCGTAATCTTATGCCTAATCACAAGTTCAGAACTGGAAGTGATTGTGAT


ASN1-S          GTCATTGCACACCTATATGAAGAACATGGAGAAGATTTTGTGGACATGCTGGATGGGATC
ASN1-T          ---------------TATGAAGAACATGGAGAAGATTTTGTGGACATGCTGGATGGGATC
ASN5-T          GTTATTGCACATCTTTATGAAGAATATGGAGAAAATTTTGTGGACATGTTGGATGGGGTG
ASN5-S          GTTATTGCACATCTTTATGAAGAATATGGAGAAAATTTTGTGGACATGTTGGATGGGGTG
                               ********* ******** ************** ******** *

ASN1-S          TTCGCTTTTGTGTTATTGGATACTCGAGATAACAGCTTTCTTGTTGCTCGTGATGCCATT
ASN1-T          TTCGCTTTTGTGTTACTGGATACTCGAGATAACAGCTTTCTTGTTGCTCGTGATGCCATT
ASN5-T          TTCTCTTTTGTATTGTTGGATACGCGCGATAACAGCTTTCTTGCTGCTCGTGATGCGATT
ASN5-S          TTCTCTTTTGTATTGTTGGATACGCGCGATAACAGCTTTCTTGCTGCTCGTGATGCAATT
                *** ******* **  ******* ** **************** ************ ***

ASN1-S          GGAATTACTTCCCTTTATATTGGTTGGGGACTTGATGGGTCTGTATGGATATCATCTGAG
ASN1-T          GGAATTACTTCCCTTTATATTGGTTGGGGACTTGATGGGTCTGTATGGATATCATCTGAG
ASN5-T          GGAATTACTCCCCTCTATATTGGTTGGGGACTTGATGGCTCTGTGTGGATTTCATCTGAG
ASN5-S          GGAATTACTCCCCTATATATTGGTTGGGGACTTGATGGCTCTGTGTGGATTTCATCTGAG
                ********* **** *********************** ***** ***** *********
```

```
ASN1-S      CTTAAGGGCTTGAATGATGACTGCGAACATTTTGAAGTTTTCCCACCAGGGCACTTGTAC
ASN1-T      CTTAAGGGCTTGAATGATGACTGCGAACATTTTGAAGTTTTCCCACCAGGACACTTGTAC
ASN5-T      CTAAAGGGCTTAAATGATGATTGTGAACATTTTGAAGTTTTCCCTCCCGGGCACTTGTAC
ASN5-S      CTAAAAGGATTAAATGGTGACTGTGAACATTTTGAAGTTTTCCCTCCCGGTCACTTGTAC
            ** ** ** ** **** *** ** ******************** ** ** *********

ASN1-S      TCTAGCAAGAATGGCGGCTTTAGGAGGTGGTACAATCCTCCTTGGTTCTCTGAGGCCATT
ASN1-T      TCTAGCAAGAATGGCGGCTTTAGGAGGTGGTACAATCCTCTTTGGTTCTCTGAGGCTATT
ASN5-T      TCGAGCAAGAATGGCGGGTTTAGGAGATGGTACAATCCTCAATGGTTCTCTGAGGCTGTT
ASN5-S      TCGAGCAAGAATGGCGGGTTTAGGAGATGGTACAATCCTCAATGGTTCTCTGAGGCTATT
            ** ************** ******** *************  **************  **

ASN1-S      CCTTCCACTCGTTATGATCCCTTAGTTCTCAGGCGTGCCTTTGAAAATGCTGTTATCAAA
ASN1-T      CCTTCCACTCCTTATGATCCCTTAGTTCTCAGGCGCGCCTTTGAAAATGCTGTTATCAAA
ASN5-T      CCATCAAATCCTTATGACCCCTTAGTTCTGAGGCGTGCCTTCGAAAATGCTGTTATTAAA
ASN5-S      CCATCAAATCCTTACGACCCCTTAGTTTTGAGACGTGCCTTCGAAAATGCTGTTATCAAA
            ** ** * ** *** ** ********* * ** ** ***** ************** ***

ASN1-S      AGGTTGATGACTGATGTCCCCTTTGGTGTTCTCCTCTCCGGGGGACTCGATTCATCCTTG
ASN1-T      AGGTTGATGACTGATGTCCCCTTTGGTGTTCTGCTCTCCGGGGGACTCGATTCATCCTTG
ASN5-T      CGGTTGATGACCGATGTACCCTTTGGTGTTCTGCTCTCCGGGGGACTTGATTCGTCTTTA
ASN5-S      CGATTGATGACCGATGTCCCCTTTGGTGTTCTGCTCTCCGGGGGACTTGATTCGTCTTTG
             * ******** ***** ************** ************** ***** ** ** 

ASN1-S      GTTGCTTCGATTACTGCTCGCTACTTGGCTGGTACAAAGGCTGCCAAGCAGTGGGGAGCA
ASN1-T      GTTGCTTCGATTACTGCCCGCTACTTGGCTGGCACAAAGGCTGCCAAGCAGTGGGGAGCA
ASN5-T      GTTGCTTCTGTCACTGCTCGCTACTTGGCTGGAACAAAAGCTGCTAAGCAATGGGGAGCG
ASN5-S      GTTGCTTCTGTCACTGCTCGCTACTTGGCTGGAACAAAAGCTGCTAAGCAATGGGGAGCA
            ********  * ***** ************** ***** ***** ***** ******** 

ASN1-S      CAGCTTCATTCCTTCTGTGTTGGCCTTGAGGGCTCACCGGATCTCAAGGCTGCAAGAGAA
ASN1-T      CAGCTTCATTCCTTCTGTGTTGGCCTTGAGGGATCACCGGATCTCAAGGCTGCAAGAGAA
ASN5-T      CAGCTTCATTCCTTCTGTGTTGGTCTCGAGGGCTCACCAGATCTCAAGGCTGCAAGAGAA
ASN5-S      CAGCTTCATTCCTTCTGTGTTGGTCTCGAGGGCTCACCAGATCTCAAGGCTGCAAGAGAA
            *********************** ** ***** ***** *********************

ASN1-S      GTTGCTGACTACTTGGGAACCGTTCACCACGAGTTTCACTTCACCGTTCAGGATGGAATT
ASN1-T      GTTGCTGACTACTTGGGAACCGTTCACCACGAGTTTCACTTCACCGTTCAGGATGGAATT
ASN5-T      GTTGCTGACTATTTGGGAACCGTTCACCACGAGTTCACCTTCACAGTTCAGGACGGAATT
ASN5-S      GTTGCTGACTATTTGGGAACCGTTCACCACGAGTTCACCTTCACAGTTCAGGATGGAATT
            *********** ***********************   ****** ******** ******

ASN1-S      GATGCAATTGAAGATGTTATTTACCATATTGAGACATACGATGTAACGACAATCAGAGCA
ASN1-T      GATGCAATTGAAGATGTTATTTACCATATTGAGACATACGATGTAACGACAATCAGAGCA
ASN5-T      GATGCTATTGAAGATGTTATTTACCATATCGAGACGTATGATGTAACAACGATCAGAGCA
ASN5-S      GATGCTATTGAAGATGTTATTTACCATATCGAGACATACGATGTAACAACGATCAGAGCA
            ***** *********************** ***** ** ******** ** *********
```

```
ASN1-S    AGCACTCCTATGTTCCTTATGTCGCGTAAGATTAAGTCACTAGGAGTGAAGATGGTCATA
ASN1-T    AGCACTCCTATGTTCCTTATGTCGCGTAAGATTAAGTCACTAGGAGTGAAGATGGTTATA
ASN5-T    AGCACCCCTATGTTCCTTATGTCGCGTAAGATTAAATCACTTGGAGTGAAGATGGTCATA
ASN5-S    AGCACTCCTATGTTCCTTATGTCGCGTAAGATTAAATCACTGGGAGTTAAGATGGTCATA
          ***** ***************************** ***** ***** ******** ***

ASN1-S    TCTGGGGAAGGATCTGATGAAGTGTTTGGTGGCTACTTGTACTTTCACAAGGCTCCCAAC
ASN1-T    TCTGGGGAAGGCTCTGATGAAGTGTTTGGTGGCTACTTGTACTTTCACAAGGCTCCCAAC
ASN5-T    TCAGGGGAAGGCTCAGATGAACTGTTTGGTGGCTACTTGTACTTCCACAAGGCTCCCAAC
ASN5-S    TCAGGGGAAGGCTCAGATGAACTGTTTGGCGGCTATTTGTACTTCCACAAGGCTCCGAAC
          ** ******** ** ****** ******* ***** ******** *********** ***

ASN1-S    AAGGAAGAGTTCCACAAGGAAACATGTCGCAAGATTAAAGCGCTTCACCAATATGACTGC
ASN1-T    AAAGAAGAGTTCCACAAGGAAACATGTCGCAAGATTAAAGCACTTCACCAATATGACTGT
ASN5-T    AAGGAAGAATTCCACACGGAGACATGTCACAAGATAAAAGCGCTTCACCAATACGACTGT
ASN5-S    AAGGAAGAATTCCATGTGGAGACATGTCACAAGATAAAAGCGCTTCACCAATACGACTGT
          ** ***** *****   *** ******* ****** ***** *********** *****

ASN1-S    TTAAGAGCAAATAAGTCAACATCTGCATGGGGTTTAGAAGCTAGAGTCCCTTTCCTAGAT
ASN1-T    TTAAGAGCAAATAAGTCAACATCTGCATGGGGCTTAGAAGCTAGAGTGCCTTTCCTAGAT
ASN5-T    TTGAGAGCAAATAAGGCAACATCAGCATGGGGCTTAGAAGCTAGAGTACCATTTCTGGAT
ASN5-S    TTGAGAGCAAATAAGGCAACATCAGCATGGGGCTTAGAAGCTAGAGTACCATTTCTGGAT
          ** ************ ******* ******** ************** ** ** ** ***

ASN1-S    AAGGAGTTCATCAATGTTGCCATGAGTATTGATCCAGAGTGGAAGTTGATTAAACCAGAG
ASN1-T    AAGGAGTTCATCAATGTTGCCATGAGTATTGATCCAGAGTGGAAGTTGATTAAACCAGAG
ASN5-T    AAAGAGTTCATCAATGTTGCTATGAGTATCGATCCTGAATGGAAGATGATTAAACACGAT
ASN5-S    AAAGAGTTCATCAACGTTGCTATGAGTATCGATCCTGAATGGAAGATGATTAAACACGAT
          ** *********** ***** ******** ***** ** ****** *********  **

ASN1-S    CAAAGGAGGATTGAAAAGTGGGCTCTAAGGAGGGCCTTTGATGATGAGGAGCATCCTTAT
ASN1-T    CAAAGGAGGATTGAGAAGTGGGCTCTAAGGAGGGCCTTTGATGATGAGGAGCATCCCTAT
ASN5-T    CAAGGTAGGATCGAGAAGTGGGTTCTTAGGAAGGCTTTTGATGATGAGGAGCACCCCTAT
ASN5-S    CATGGTAGGATCGAGAAGTGGGTTCTTAGGAAGGCTTTTGATGATGAGGAGCAACCCTAT
          **  * ***** ** ******* *** **** *** ***************** ** ***

ASN1-S    CTCCCAAAGCACATCCTGTATAGGCAAAAAGAACAATTCAGTGATGGCGTGGGCTATAGT
ASN1-T    CTCCCAAAGCACATCCTATACAGGCAGAAAGAACAATTCAGTGATGGCGTAGGCTATAGT
ASN5-T    CTCCCAAAGCATATTTTGTACCGGCAGAAAGAACAATTCAGCGATGGTGTAGGCTATAGT
ASN5-S    CTCCCAAAGCATATTCTGTACCGGCAGAAAGAACAATTCAGTGATGGCGTAGGCTATAGT
          *********** **  * **  **** ************** ***** ** *********

ASN1-S    TGGATAGATGGACTCAAAGCACATGCTGAACAACATGTGACCAATAGGATGATGTTTAAT
ASN1-T    TGGATAGATGGACTCAAAGCACATGCTGAACAACATGTGACCAATAGGATGATGCTTAAT
ASN5-T    TGGATTGATGGGCTCAAAGCACATGCTGAACAACATGTGACTGATAGGATGATGCTTAAT
ASN5-S    TGGATCGATGGACTCAAAGCACATGCTGAACAACATGTGACTGATAGGATGATGCTTAAT
          ***** ***** *****************************  *********** *****

ASN1-S    GCTTCACATATATTCCCTCATAACACACCCATTACAAAGGAAGCATACTACTATAGGATG
ASN1-T    GCTTCACATATATTCCCTCATAACACACCGATTACAAAGGAAGCATACTATTATAGGATG
ASN5-T    GCTTCACATATCTTCCCTCACAACACTCCAACTACAAAGGAAGCATACTATTACAGGATG
ASN5-S    GCTGCACATATCTTCCCTCACAACACTCCAACTACAAAGGAAGCATACTATTACAGGATG
          *** ******* ******** ***** ** * ****************** ** ******
```

```
ASN1-S      ATTTTCGAGCGCTTTTTCCCACAGAATTCAGCTGGGCTAACCGTTCCTGGAGGAGCAAGT
ASN1-T      ATTTTCGAGCGCTTTTTCCCACAGAATTCAGCTGGGCTAACCGTTCCTGGAGGAGCGAGT
ASN5-T      ATTTTTGAGAGGTTCTTTCCACAGAATTCAGCAAGGCTAACTGTTCCTGGAGGACCGAGT
ASN5-S      ATTTTCGAGAGGTTCTTCCCACAGAATTCAGCAAGGCTAACTGTTCCTGGAGGACCGAGT
            ***** *** * ** ** **************  ******* ************ * ***

ASN1-S      GTGGCGTGTAGCACAGCTAAAGCTGTAGAGTGGGATGCTTCTTGGTCAAAGAACCTTGAT
ASN1-T      GTGGCGTGTAGCACAGCTAAAGCTGTAGAGTGGGATGCTTCTTGGTCAAAGAACCTTGAT
ASN5-T      ATAGCTTGCAGCACGGCTAAAGCTATTGAGTGGGACGCTTCTTGGTCGAACAACCTTGAT
ASN5-S      ATAGCTTGCAGCACAGCTAAAGCTATTGAGTGGGATGCTTCGTGGTCGAACAACCTTGAT
             * ** ** ***** ********* * ******** ***** ***** ** *********

ASN1-S      CCTTCAGGCAGGGCTGCTATTGGTGTACATAACTCGGCTTATGAGAATCATGTACCTGCT
ASN1-T      CCTTCAGGAAGGGCTGCTATTGGTGTACATAACTCAGCTTATGAGAATCATGAACCTGCT
ASN5-T      CCTTCCGGTAGGGCTGCTATCGGTGTACATAACTCGGCTTATGACGATCATCTACCCGAT
ASN5-S      CCTTCCGGTAGGGCTGCAATCGGTGTACATAACTCGGCTTATGACGATCATCTCCCCGAT
            ***** ** ******** ** ************** ********  *****   ** * *

ASN1-S      ATGGCTAATGGGAATTTGACCAAAAAAATCATTGGTCGTGTGCCTTCTATGGTAGAAGTT
ASN1-T      ATGGCTAATGGGAATTTGGCCACAAAAATCATTGGCCGTGCGCCGTCTATGGTAGAAGTT
ASN5-T      GTTGGTAATGGGAATTTGGACACAACGATCATCGATAATGTGCCAAGGATGGTAGGAGTG
ASN5-S      GTTGGTAATGGGAATTTGGACACAACGATCATCGATAATGTGCCGAGGATGGTAGGAGTG
             * * *************  ** **  ***** *    ** ***    ******* ***

ASN1-S      GGTGCTGCTCCCGAGCTCACAATAAAGAGTTAG
ASN1-T      GGTGCTGCTCATGAGCTCACAATAAGGAGTTAG
ASN5-T      GGTGCTTCTGCAGAGCTCACAATAAGGAGCTAG
ASN5-S      GGTGCTGCTGCAGAGCTCACAATAAGGAGCTAG
            ****** **   ************* *** ***
```

(b)

```
ASN5-T          MCGILALLGCPDDSQAKRVRVLELSRRLKHRGPDWSGIYQHGDFYLAHQRLAIIDPTSGD
ASN5-S          MCGILALLGCSDDSQAKRVRVLELSRRLKHRGPDWSGIYQHGDFYLAHQRLAIIDPASGD
ASN1-S          MCGILAVLGCSDDSQAKRVRVLELSRRLKHRGPDWSGLYQHGDCYLAHQRLAIVDPASGD
ASN1-T          MCGILAVLGCSDDSQAKRVRVLELSRRLKHRGPDWSGLYQHGDCYLAHQRLAIVDPASGD
                ******:***.**************************:***** *********:**:***

ASN5-T          QPLFNQDKTIVVTVNGEIYNHEKLRNLMPNHKFRTGSDCDVIAHLYEEYGENFVDMLDGV
ASN5-S          QPLFNQDKTIVVTVNGEIYNHEKLRNLMPNHKFRTGSDCDVIAHLYEEYGENFVDMLDGV
ASN1-S          QPLFNEDKTIVVTVNGEIYNHEQLRKQMPNHKFRTGSDCDVIAHLYEEHGEDFVDMLDGI
ASN1-T          QPLFNEDKTIVVT--------------------------------YEEHGEDFVDMLDGI
                *****:*******                                ***:**:*******:

ASN5-T          FSFVLLDTRDNSFLAARDAIGITPLYIGWGLDGSVWISSELKGLNDDCEHFEVFPPGHLY
ASN5-S          FSFVLLDTRDNSFLAARDAIGITPLYIGWGLDGSVWISSELKGLNGDCEHFEVFPPGHLY
ASN1-S          FAFVLLDTRDNSFLVARDAIGITSLYIGWGLDGSVWISSELKGLNDDCEHFEVFPPGHLY
ASN1-T          FAFVLLDTRDNSFLVARDAIGITSLYIGWGLDGSVWISSELKGLNDDCEHFEVFPPGHLY
                *:************.********.*********************.**************

ASN5-T          SSKNGGFRRWYNPQWFSEAVPSNPYDPLVLRRAFENAVIKRLMTDVPFGVLLSGGLDSSL
ASN5-S          SSKNGGFRRWYNPQWFSEAIPSNPYDPLVLRRAFENAVIKRLMTDVPFGVLLSGGLDSSL
ASN1-S          SSKNGGFRRWYNPPWFSEAIPSTRYDPLVLRRAFENAVIKRLMTDVPFGVLLSGGLDSSL
ASN1-T          SSKNGGFRRWYNPLWFSEAIPSTPYDPLVLRRAFENAVIKRLMTDVPFGVLLSGGLDSSL
                ************* *****:**. ************************************

ASN5-T          VASVTARYLAGTKAAKQWGAQLHSFCVGLEGSPDLKAAREVADYLGTVHHEFTFTVQDGI
ASN5-S          VASVTARYLAGTKAAKQWGAQLHSFCVGLEGSPDLKAAREVADYLGTVHHEFTFTVQDGI
ASN1-S          VASITARYLAGTKAAKQWGAQLHSFCVGLEGSPDLKAAREVADYLGTVHHEFHFTVQDGI
ASN1-T          VASITARYLAGTKAAKQWGAQLHSFCVGLEGSPDLKAAREVADYLGTVHHEFHFTVQDGI
                ***:************************************************ *******

ASN5-T          DAIEDVIYHIETYDVTTIRASTPMFLMSRKIKSLGVKMVISGEGSDELFGGYLYFHKAPN
ASN5-S          DAIEDVIYHIETYDVTTIRASTPMFLMSRKIKSLGVKMVISGEGSDELFGGYLYFHKAPN
ASN1-S          DAIEDVIYHIETYDVTTIRASTPMFLMSRKIKSLGVKMVISGEGSDEVFGGYLYFHKAPN
ASN1-T          DAIEDVIYHIETYDVTTIRASTPMFLMSRKIKSLGVKMVISGEGSDEVFGGYLYFHKAPN
                ***********************************************:************

ASN5-T          KEEFHTETCHKIKALHQYDCLRANKATSAWGLEARVPFLDKEFINVAMSIDPEWKMIKHD
ASN5-S          KEEFHVETCHKIKALHQYDCLRANKATSAWGLEARVPFLDKEFINVAMSIDPEWKMIKHD
ASN1-S          KEEFHKETCRKIKALHQYDCLRANKSTSAWGLEARVPFLDKEFINVAMSIDPEWKLIKPE
ASN1-T          KEEFHKETCRKIKALHQYDCLRANKSTSAWGLEARVPFLDKEFINVAMSIDPEWKLIKPE
                ***** ***.***************:*****************************:** :

ASN5-T          QGRIEKWVLRKAFDDEEHPYLPKHILYRQKEQFSDGVGYSWIDGLKAHAEQHVTDRMMLN
ASN5-S          HGRIEKWVLRKAFDDEEQPYLPKHILYRQKEQFSDGVGYSWIDGLKAHAEQHVTDRMMLN
ASN1-S          QRRIEKWALRRAFDDEEHPYLPKHILYRQKEQFSDGVGYSWIDGLKAHAEQHVTNRMMFN
ASN1-T          QRRIEKWALRRAFDDEEHPYLPKHILYRQKEQFSDGVGYSWIDGLKAHAEQHVTNRMMLN
                : *****.**.******:************************************:***:*

ASN5-T          ASHIFPHNTPTTKEAYYYRMIFERFFPQNSARLTVPGGPSIACSTAKAIEWDASWSNNLD
ASN5-S          AAHIFPHNTPTTKEAYYYRMIFERFFPQNSARLTVPGGPSIACSTAKAIEWDASWSNNLD
ASN1-S          ASHIFPHNTPITKEAYYYRMIFERFFPQNSAGLTVPGGASVACSTAKAVEWDASWSKNLD
ASN1-T          ASHIFPHNTPITKEAYYYRMIFERFFPQNSAGLTVPGGASVACSTAKAVEWDASWSKNLD
                *:******** ******************** ******.*:*******:*******:***

ASN5-T          PSGRAAIGVHNSAYDDHLPDVGNGNLDTTIIDNVPRMVGVGASAELTIRS
ASN5-S          PSGRAAIGVHNSAYDDHLPDVGNGNLDTTIIDNVPRMVGVGAAAELTIRS
ASN1-S          PSGRAAIGVHNSAYENHVPAMANGNLTKKIIGRVPSMVEVGAAPELTIKS
ASN1-T          PSGRAAIGVHNSAYENHEPAMANGNLATKIIGRAPSMVEVGAAHELTIRS
                **************::* * :.**** ..**...* ** ***: ****.*
```

Figure S5. Nucleotide and amino acid sequences of *Nicotiana tabacum* ASN1 and ASN5. (**a**) Coding sequence alignments for *ASN1* and *ASN5*. (**b**) Amino acid sequence alignments for ASN1 and ASN5. * indicates identical residues.

(a) (b) (c)

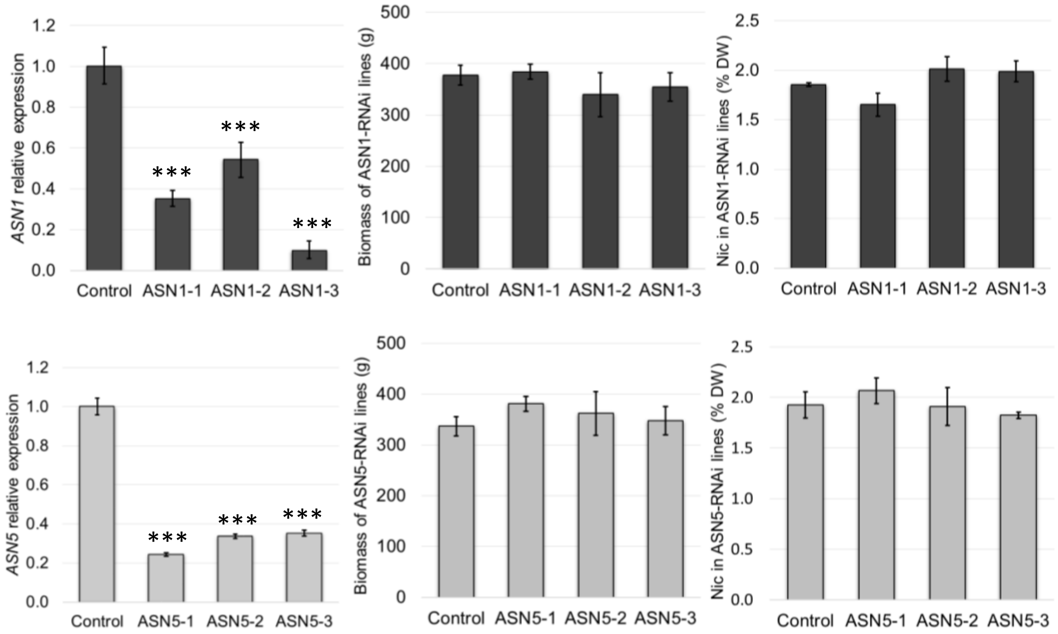

Figure S6. (a) *ASN1* and *ASN5* transcript levels in burley tobacco leaves. The relative expression levels of *ASN1* and *ASN5* transcripts were estimated by qPCR with RNA isolated from lower leaves after 3 days of curing. Data were collected from four plants. These data are summarised in histogram bars, in which each bar signifies the expression levels of *ASN1* and *ASN5* in ASN-RNAi plants relative to the wild-type (WT, control), and the error bar represents the SD. Asterisks indicate statistical significance when comparing WT control plants to ASN-RNAi plants ($n = 4$; ***$p < 0.001$; t-test). **(b)** Biomass of tobacco leaf tissues during growth (green stage; middle leaf position). Data were collected from four biological replicates ($n = 4$). These data are summarised in histogram bars, in which each bar signifies the weight of leaf tissue in grams (g), and the error bar represents the SD. Data were analysed by the t-test, and no significant difference was observed between the RNAi lines and WT plants. **(c)** Nicotine content (Nic) in tobacco leaf tissues. Data were collected from four biological replicates. These data are summarised in histogram bars, in which each bar signifies the percentage of nicotine (% dry weight [DW]), and the error bar represents the SD. Data were analysed by the t-test, and no significant difference was observed between the RNAi lines and WT plants.
